# Supplementary material for: Association of Clonal Hematopoiesis of Indeterminate Potential with Inflammatory Gene Expression in Patients with COPD
Source: Cells. 2022 Jul 5;11(13):2121. doi: 10.3390/cells11132121 (PMC9265467; doi:10.3390/cells11132121)
Supplement: Supplementary file 1 [file cells-11-02121-s001.zip › cells-1744429-supplementary.pdf]

cells MDPI

Supplementary Material

Association of Clonal Hematopoiesis of Indeterminate Potential with Inflammatory Gene Expression in Patients With COPD: Online Supplementary File

1. Study population

COSYCONET is a German multicenter prospective observational trial, which recruited 2741 patients aged 40 years and older with diagnosis of COPD between 2010 and 2013 in 31 study centers. COSYCONET was approved by the ethics committees at all participating sites, and all participants provided written consent. The study is registered at ClinicalTrials.gov (NCT01245933). The Study protocol V1.6 from the 23.05.2011 states full compliance with national laws, ICH Guideline for Good Clinical Practice (GCP) E6 from june 1996 and the declaration of Helsinki.'

The study subjects of COSYCONET gave written consent upon study inclusion for genetic analysis of blood samples. Participants also gave written consent, that results genetic analysis would not be reported to them. All blood samples for CHIP sequencing were drawn at visit 1 (between 2011 and 2013). We estimate the risk of malignancy to be 0.5-1% per year in CHIP positive patients. DNA sequencing and data analysis took place 6-8 years after sampling, the study team considered this no longer relevant for the patient's safety. GOLD 0 refers to patients with smoking history and chronic bronchitis who do not fulfill the spirometric criterion FEV1/FVC <0.7. We examined the effects of CHIP in patients with early COPD (symptomatic individuals with smoking history) with FEV1/FVC-ratio >70% (GOLD 0), in patients with mild, moderate and severe COPD (FEV1 80 – 30% as in GOLD stages I-III) and patients with severe or very severe COPD (FEV1<50% as in GOLD stage III and IV).

Known haematologic disorders or malignancy was not an exclusion criteria for entering COSYCONET. However, differential blood count was available for 84% of patients. There was one case of thrombocyteamia (44/) without further changes in other cell lines. There were no cases of severe anemia or polyglobulinaemia (Hb 8,5-18,9), in 6 cases hematocrit exceeded 50% (maximum 55%). No significant leukocytosis occurred (leucocytes 4,79 – 18,39 giga/l).

2. DNA methylation analysis

DNA was isolated from the EDTA blood samples obtained from COSYCONET using the QIAamp DNA mini Kit (Qiagen), according to the manufactures protocol. DNA concentration was quantified using the Qubit dsDNA HS Assay kit (Q32851, Invitrogen). 200–500 ng of DNA was used as input for DNA methylation analysis. The Infinium HumanMethylation EPIC BeadChip (850k) (WG-317, Illumina) was used to determine the DNA methylation status of more than 850.000 CpG sites, respectively following the producer's guidelines [14]. On-chip quality metrics of all samples were carefully controlled.

Raw intensities derived from IDAT files passed quality control on probes and samples. Probes and samples have been filtered for SNP-enrichment and highest impurity using Greedycut (FDR adjusted $p < 0.05$), missing values and sex chromosomes. Individual site-based methylation levels were calculated and subsequently used for region-based methylation level assessment (CpG island/promoter/ tails/gene body).

3. Sample Preparation for NGS and High-Throughput Sequencing

Before sequencing, the pooled libraries were diluted and denatured according to the NextSeq System Denature and Dilute Libraries Guide (Illumina) and 1% PhiX DNA was added. The pooled libraries were sequenced on a NextSeq 500 sequencer (Illumina) using the NextSeq 500/550 Mid Output, version 2 kit (300 cycles) according to the manufacturer's instructions. Briefly, the sequencer was operated in a paired-end sequencing mode with 2 × 150 bp read length and 2 × 8 bp index read length. The BCL files were demultiplexed and converted to FASTQ files using the FASTQ Generation tool on BaseSpace (Illumina). The median coverage across all BMC samples was 4282× before UMI family clustering and 630× with inclusion of UMIs.

Variant Calling and Annotation Strategies: Read quality was assessed using FastQC. FASTQ files from each patient were merged and reads were grouped into unique molecular identifier (UMI) families using the UMI-tools software package. Reads were mapped to the hg19 draft of the human genome using Burrows-Wheeler Alignment–MEM. The `dedup` command of the UMI-tools software package was used to remove polymerase chain reaction duplicates with the same UMIs and alignment coordinates. Variant calling was performed using FreeBayes without allele frequency threshold, a minimum alternate read count of 2, and a minimum base and mapping quality of 20. Variant effect prediction and variant annotation was performed using SnpEff and SnpSift [9,15,16].

The identified variants were processed and filtered using the R programming language, version 3.3.1 (R Programming). Common single-nucleotide polymorphisms with a minor allele frequency of at least 5% in either the 1000 Genome Project, Exome Variant Server, or ExAC databases were excluded. In addition, variants with a low mapping quality (<20) and variants occurring in 8% or more of the patients in the studied cohort were considered as technical artifacts and excluded. Furthermore, variants covered with fewer than 100 reads in at least 1 set of amplicons (CAT A or CAT B), variants called with only 1 of the set of amplicons (CAT A or CAT B), single-nucleotide polymorphisms identified as common in the single-nucleotide polymorphism database (≥1% in the human population), and variants with sequence ontology terms "LOW" or "MODIFIER" were filtered out. According to previously established definitions, all variants with a variant allele fraction (VAF) of at least 0.02 (2%) were considered; VAF was calculated by using the formula VAF = alternate reads / (reference + alternate reads). Variants with a VAF of 0.45 to 0.55 were not considered to exclude potential germline variants. The variants were further validated on the basis of being reported in the literature and/or the Catalogue of Somatic Mutations in Cancer (https://cancer.sanger.ac.uk/cosmic) and ClinVar (https://www.ncbi.nlm.nih.gov/clinvar).

4. Metabolomic and cytokine analysis

Plasma samples were isolated during study visits at the according study center. For isolation and direct processing of samples the studies (SOP) was followed. Blood vials were centrifuged and frozen within 1h after sampling. A temperature log protocol was followed. All biomaterials were stored in the central biobank. The transfer of the samples from the study centers to the central biobank and all related procedures are defined by SOPs. Briefly, the samples are sent every four weeks after previous announcement to the central biobank. The transportation system was established, and every step was described in the corresponding SOP. The samples were transported under dry ice pellets by an overnight express service. After receipt at the biobank the samples were inspected immediately, imported in the database and stored at -80°C until further processing. Oxylipins and choline pathway metabolites were extracted from plasma by adding 3 x the volume of ethyl acetate and 4 x the volume of methanol, respectively. Samples were vortexed, centrifuged and either the upper layer (oxylipins) or supernatant (cholines) were dried down, resuspended in solvent, and analyzed in an Agilent 1290 Infinity LC system coupled to a

Sciex QTrap 5500 mass spectrometer in the negative (oxylipins) or positive (cholines) ionization modes. TNF-α and IL-6 were analyzed using ELISA kits.

5. Cell culture and gene expression analysis by qPCR

Human macrophages were generated from peripheral blood mononuclear cells (PBMCs) as previously described [17,18]. Briefly, PBMCs isolated from buffy coats obtained from the blood bank of the Universities of Giessen and Marburg Lung Center using Ficoll density gradient centrifugation. Platelets and red blood cells (RBC) were removed by two washing steps with RBC lysis buffer (BD Biosciences) and phosphate-buffered saline (PBS), respectively. Finally, monocytes were differentiated to macrophages during 10 days in the macrophage complete media, which is RPMI 1640 medium containing 2.5% (v/v) human serum, 4 mM L-glutamine, and 1% (v/v) penicillin/streptomycin in six-well tissue culture plates. Macrophages were transfected with stealth RNAi siRNAs against DNMT3A (HSS176225) or a scramble negative control with medium GC content (Thermofisher) using the HiPerFect Transfection Reagent (Qiagen) in an optimum serum-free medium based on the manufacturer's protocol.

Total RNA was extracted with RNeasy Mini Kit (Qiagen, Hilden, Germany) which then transcribed into complementary DNA (cDNA) using the high-capacity cDNA reverse transcription Kit (Applied Biosystems, Waltham, USA) according to the manufacturer's instructions. Also, quantitative PCR (qPCR) was performed using SYBR Green PCR Master mix and the StepOne real-time PCR System (Applied Biosystems, Waltham, USA) at the following conditions: 10 min at 95°C, followed by 40 cycles of 30s at 95°C, 30s at 58° to 60°C. Analysis was done using the StepOne plus software and GraphPad Prism. Expression was determined using the ΔCT method. CT-values were normalized to the housekeeping gene-encoding hypoxanthine-guanine phosphoribosyl transferase (*HPRT1*) using the equation $\Delta CT = CT_{reference} - CT_{target.}$ The primer sequences used in this study are shown in table S2.

6. Human COPD Precision Cut Lung Slides (PCLS)

Low melt agarose (2%; 6351; Carl Roth GmbH + Co. KG, Karlsruhe, Germany) was liquefied in RPMI 1640 medium (P04-16500; PAN-Biotech GmbH, Aidenbach, Germany) containing 10% fetal bovine serum (FBS; F4135; Merck KGaA, Darmstadt, Germany) and 1% penicillin-streptomycin (15070-063; GIBCO™, Thermo Fisher Scientific Inc., Waltham, MA, USA). A piece of the human lung was inflated with lukewarm low melt agarose solution through bronchi and left in ice-cold phosphate-buffered saline (PBS; P04-36503, PAN-Biotech, Aidenbach, Germany) solution until the agarose solidified. The PCLS were cut 400 µm thick using a vibratome (Microm HM650V, Thermo Fisher Scientific Inc., Waltham, MA, USA) and cultured in RPMI 1640 medium containing 10%, 1% penicillin-streptomycin, 100 µg/ml Normocin™ (ant-nt-1; InvivoGen, Toulouse, France). The medium was changed hourly in the first 3 hours to remove the dead cells. PCLS were then cryopreserved using freezing medium (FBS 50%, RPMI 1640 medium 40%, and dimethyl sulfoxide 10%) and stored in liquid nitrogen until further use. Before the treatment, PCLS were defrozen in RPMI 1640 medium (containing 10%, 1% penicillin-streptomycin, and 100 µg/ml Normocin™) and placed in an incubator for 24 hours to recover. PCLS were then treated with conditional media of a DNMT3A-deficient monocyte cell line (THP1) for 48 hours. Afterward, the PCLS were washed with ice-cold PBS and homogenized in commercially available protein lysis buffer (9803; Cell Signaling Technology Inc., Danvers, MA, USA) containing 1 mM phenylmethylsulfonyl fluoride (PMSF), using a Precellys® 24 bead beating tissue homogenizer (Bertin Corp; Thermo Fisher Scientific Inc., Waltham, MA, USA) and following the manufacturer´s recommendation. Protein concentration in the tissue lysates was determined using the Bradford assay (#5000113-5; Bio-Rad Laboratories GmbH, Hercules, CA, USA) following the manufacturer´s recommendation.

THP-1 cells seeded in 6-well plates were stimulated with 100 ng/ml of phorbol 12-myristate 13-acetate (PMA) for 24 hrs. Then the media was removed and cells were

washed once with complete media without PMA and keep in culture for further 24 hrs in complete media in the absence of PMA. Cells were then transfected with stealth RNAi siRNAs against DNMT3A (HSS176225) or a scramble negative control with medium GC content (Thermofisher) by using Lipofectamine™ 3000 (Invitrogen) according to the manufacturer's instructions.

7. Western Blotting

The lysate was cleaned through high-speed centrifugation. Proteins were separated using 10% polyacrylamide gels and transferred to nitrocellulose membrane. Following the blocking with 5% milk, the membranes were incubated with a primary antibody overnight at 4°C. After washing with tris-buffered saline containing Tween 20 for 3 times, the blots were incubated with secondary antibodies coupled with horse radish peroxidase. The protein-antibody conjugates were detected using an enhanced chemiluminescence detection system. Image reader (Fujifilm) was used for visualizing and quantifying western blot bands. Expression was quantified using band intensity values (in arbitrary units) that were normalized to GAPDH. Uncropped images of all western blots are provided in the supplementary file. The Western blots shown in the figure are representative of 3 independent experiments. Antibody details were shown in table S3.

References

14. Tombor, L.S.; John, D.; Glaser, S.F., Luxán, G.; Forte, E.; Furtado, M.; Rosenthal, N.; Baumgarten, N.; Schulz, M.H.; Wittig, J. et al. Single cell sequencing reveals endothelial plasticity with transient mesenchymal activation after myocardial infarction. *Nat. Commun.* **2021**, *12*(1), 681. https://doi.org/10.1038/s41467-021-20905-1.
15. Mas-Peiro, S.; Hoffmann, J.; Fichtlscherer, S.; Dorsheimer, L.; Rieger, M.A.; Dimmeler, S.; Vasa-Nicotera, M.; Zeiher, A.M.; Clonal haematopoiesis in patients with degenerative aortic valve stenosis undergoing transcatheter aortic valve implantation. *Eur. Heart, J.* **2020**, *41*(8), 933–939. https://doi.org/10.1093/eurheartj/ehz591.
16. Ortmann, C.A.; Dorsheimer, L.; Abou-El-Ardat, K.; Hoffrichter, J.; Assmus, B.; Bonig, H.; Scholz, A.; Pfeifer, H.; Martin, H.; Schmid, T. et al. Functional Dominance of CHIP-Mutated Hematopoietic Stem Cells in Patients Undergoing Autologous Transplantation. *Cell Rep*. **2019**, *27*(7), 2022–2028. https://doi.org/10.1016/j.celrep.2019.04.064.
17. Sarode, P.; Zheng, X.; Giotopoulou, G.A.; Weigert, A.; Kuenne, C.; Günther, S.; Friedrich, A.; Gattenlöhner, S.; Stiewe, T.; Brüne, B. et al. Reprogramming of tumor-associated macrophages by targeting β-catenin/FOSL2/ARID5A signaling: A potential treatment of lung cancer. *Sci. Adv.* **2020**, *6*(23), 6105. https://doi.org/10.1126/sciadv.aaz6105.
18. Zheng X, Weigert A, Reu S, Guenther S, Mansouri S, Bassaly B, Gattenlöhner S, Grimminger F, Pullamsetti S, Seeger, W., Winter, H., Savai, R. Spatial Density and Distribution of Tumor-Associated Macrophages Predict Survival in Non-Small Cell Lung Carcinoma. *Cancer Res*. **2020**, *80*(20), 4414–4425. https://doi.org/10.1158/0008-5472.CAN-20-0069

Supplementary Figure

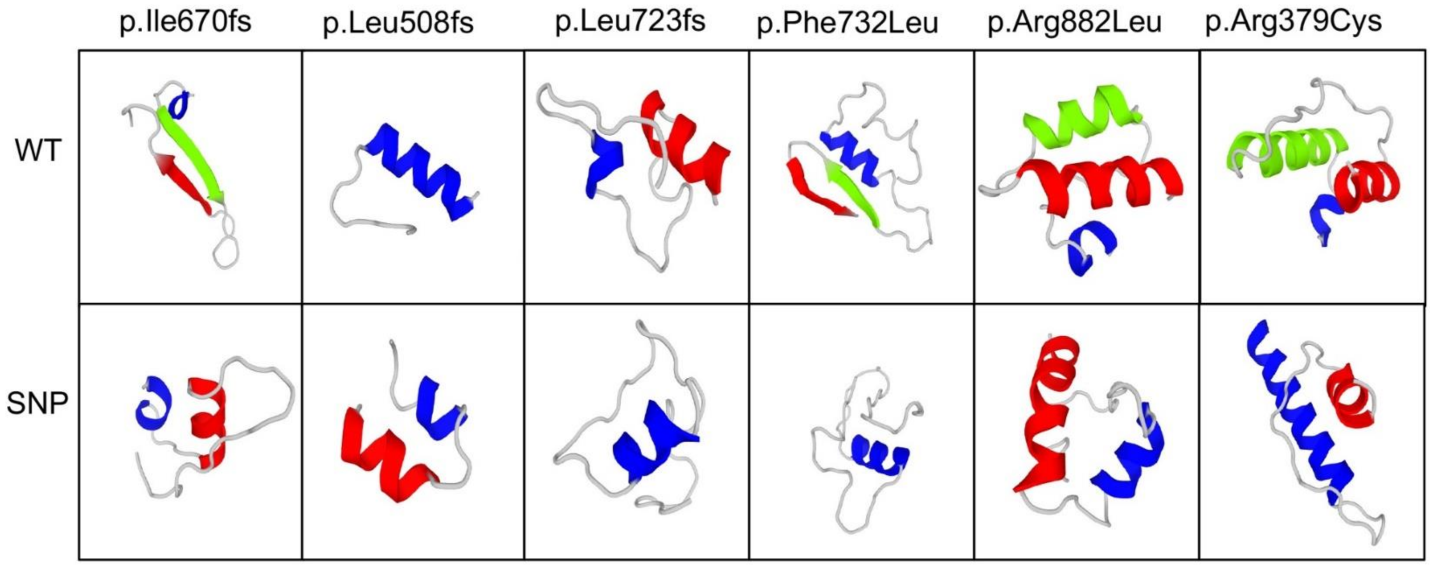

Figure S1. In silico analysis of DNMT3A Evaluation of single-nucleotide polymorphism (SNP) effect on the predicted DNMT3A structure by homology modeling. Upper panel: Structure of human DNMT3A structure at the specific amino acids mentioned on the top (marked as WT). Lower panel: Predicted tridimensional DNMT3A including the frameshift (fs) in the Ile670, Leu508, Leu723 and the substitution in the Phe732 (Phe732Leu), Arg882 (Arg882Leu), Arg379 (Arg379Cys).

Supplementary Tables

Table S1. List of genes analyzed by the custom TruSeq targeted sequencing panel.

ABL1	*CBL*	*DNMT3A*	*GNAS*	*JAK3*	*NOTCH1*	*PTPN11*	*SRSF2*
ASXL1	*CBLB*	*ETV6/TEL*	*GNB1*	*KDM6A*	*NPM1*	*RAD21*	*STAG2*
ATRX	*CBLC*	*EZH2*	*HRAS*	*KIT*	*NRAS*	*RUNX1*	*TET2*
BCOR	*CDKN2A*	*FBXW7*	*IDH1*	*KRAS*	*PDGFRA*	*SETBP1*	*TP53*
BCORL1	*CEBPA*	*FLT3*	*IDH2*	*MLL*	*PHF6*	*SF3B1*	*U2AF1*
BRAF	*CSF3R*	*GATA1*	*IKZF1*	*MPL*	*PPM1D*	*SMC1A*	*WT1*
CALR	*CUX1*	*GATA2*	*JAK2*	*MYD88*	*PTEN*	*SMC3*	*ZRSR2*

Table S2. qPCR primer list.

**Gene**		**Sequence (5`–3`)**
HPRT	FP	TGACACTGGCAAAACAATGCA
	RP	GGTCCTTTTCACCAGCAAGCT
DNMT3A	FP	CGAGTCCAACCCTGTGATGATTG
	RP	GCTGGTCTTTGCCCTGCTTTATG
TNFα	FP	GAGGCCAAGCCCTGGTATG
	RP	CGGGCCGATTGATCTCAGC
IL6	FP	AGCCAGAGCTGTGCAGATGAG
	RP	TGGCATTTGTGGTTGGGTC

Table S3. List of primary antibodies.

**Antibody**	**Catalog number**	**Company**
GAPDH	MA5-15738	Invitrogen
Anti-IL6	ab9324	Abcam
Anti-TNF alpha	ab6671	Abcam
Anti-IL1β	12703	Cell Signaling

Table S4. DNMT3A SNPs and List of CHIP-associated somatic variants identified in COPD patients:.

Patient ID	Gene	Position	Mutation cDNA	Mutation protein	VAF	Variant type	Variant classification	Ref. Allele	Alt. Allele	Ref. Reads (CAT A)	Alt. Reads (CAT A)	Ref. Reads (CAT B)	Alt. Reads (CAT B)
1	*DNMT3A*	Chr2: 25467408	c.1667+1G>T		0.1048	SNP	splice donor variant Intron variant	C	A	353	52	181	16
2	*DNMT3A*	Chr2: 25463289	c.2204A>G	p.Tyr735Cys	0.0295	SNP	missense variant	T	C	1627	60	2659	64
3	*DNMT3A*	Chr2: 25457192	c.2695C>T	p.Arg899Cys	0.0405	SNP	protein protein contact missense variant	G	A	2429	85	7851	389
4	*DNMT3A*	Chr2: 25464525	c.1988C>T	p.Ser663Leu	0.2415	SNP	missense variant	G	A	660	173	150	57
5	*DNMT3A*	Chr2: 25464505	c.2007delC	p.Ile670fs	0.0414	DEL	frameshift variant	TG	T	3104	126	2033	94
6	*DNMT3A*	Chr2: 25464439	c.2074C>T	p.Gln692*	0.0840	SNP	stop gained	G	A	1409	120	914	90
7	*DNMT3A*	Chr2: 25468152	c.1523delT	p.Leu508fs	0.0450	DEL	frameshift variant	GA	G	8693	475	1727	69
8	*DNMT3A*	Chr2: 25469646	c.1123-1G>T		0.0625	SNP	splice acceptor variant intron variant	C	A	439	39	197	9
9	*DNMT3A*	Chr2: 25463299	c.2194T>C	p.Phe732Leu	0.0994	SNP	missense variant	A	G	677	67	621	76
10	*DNMT3A*	Chr2: 25457242	c.2645G>T	p.Arg882Leu	0.0378	SNP	missense variant	C	A	6546	259	12199	479
11	*DNMT3A*	Chr2: 25470607	c.866delG	p.Gly289fs	0.0288	DEL	frameshift variant	GC	G	8108	242	4870	147
12	*DNMT3A*	Chr2: 25463519	c.2162dupA	p.Leu723fs	0.0318	INS	frameshift variant	C	CT	811	30	1518	44
13	*DNMT3A*	Chr2: 25470516	c.958C>T	p.Arg320*	0.0354	SNP	stop gained	G	A	12716	542	9857	306
14	*DNMT3A*	Chr2: 25467436	c.1640T>A	p.Leu547His	0.0212	SNP	missense variant	A	T	724	17	402	8
15	*DNMT3A*	Chr2: 25469633	c.1135C>T	p.Arg379Cys	0.0280	SNP	missense variant	G	A	475	14	213	6
16	*DNMT3A*	Chr2: 25470588	c.886G>C	p.Val296Leu	0.0207	SNP	missense variant	C	G	4791	105	5744	118
17	*DNMT3A*	Chr2: 25470582	c.892G>A	p.Gly298Arg	0.0282	SNP	missense variant	C	T	662	16	618	21
18	*DNMT3A*	Chr2: 25463566	c.2116G>A	p.Gly706Arg	0.0199	SNP	protein protein contact missense variant	C	T	277	6	626	12
19	*DNMT3A*	Chr2: 25459803	c.2478+2T>G		0.0811	SNP	splice donor variant intron variant	A	C	5302	260	804	105
20	*DNMT3A*	Chr2: 25457182	c.2705T>G	p.Phe902Cys	0.3368	SNP	missense variant	A	C	468	239	544	276

Abbreviations: DNMT3A – DNA methyl transferase 3A; SNP – single nucleotide polymorphism; VAF – Variant allele fraction; Ref – reference; Alt – alternate; DEL – deletion; INS – insertion. *:amino acid unknown.

Table S5. Methylation level of inflammatory markers.

ID	Chr.	Start	End	Symbol	EntrezID	Methylation level in DNMT3A-chip	Methylation level in Non-chip	Difference in Methylation level	comb.p.adj.fdr	combinedRank
ENSG00000136244	chr7	22765503	22771621	*IL6*	3569	0.4453	0.446	-0.001	0.487	27638
ENSG00000232810	chr6	31543344	31546113	*TNF*	7124	0.462	0.4612	0.001	0.487	27078
ENSG00000125538	chr2	1,14E+08	1,14E+08	*IL1B*	3553	0.397	0.421	-0.024	0.487	4061
ENSG00000169429	chr4	74606223	74609433	*IL8*	3576	0.340	0.342	-0.001	0.487	28597
